# Dr. Edward Little

**Published:** 1902-12

**Authors:** 


Dr. Edward Little, of Buffalo, died at his home, 348 Virginia Street, October 29, 1902, aged 75 years. Dr. Little graduated in medicine at the University of Buffalo in 1862. For many years past he had been conducting a drug store under the Mansion House and attended to that business until within three weeks of his death. He is survived by three children, Miss Elizabeth G. Little, Mrs. E. C. Beale and Dr. William R. Little, all of Buffalo.

